# Effect of Administration of Azithromycin and/or Probiotic Bacteria on Bones of Estrogen-Deficient Rats

**DOI:** 10.3390/ph15080915

**Published:** 2022-07-24

**Authors:** Urszula Cegieła, Piotr Londzin, Aleksandra Janas, Maria Pytlik, Joanna Folwarczna

**Affiliations:** Department of Pharmacology, Faculty of Pharmaceutical Sciences in Sosnowiec, Medical University of Silesia, Katowice, Jagiellońska 4, 41-200 Sosnowiec, Poland; ucegiela@sum.edu.pl (U.C.); ajdna@op.pl (A.J.); mariapytlik@gmail.com (M.P.); jfolwarczna@sum.edu.pl (J.F.)

**Keywords:** azithromycin, probiotic bacteria, *Lactobacillus rhamnosus*, skeletal system, rat, ovariectomy, osteoporosis

## Abstract

The gut microbiota plays an important role in maintaining homeostasis, including that of the skeletal system. Antibiotics may affect the skeletal system directly or indirectly by influencing the microbiota. Probiotic bacteria have been reported to favorably affect bones in conditions of estrogen deficiency. The aim of this study was to investigate the effects of azithromycin (AZM) administered alone or with probiotic bacteria (*Lactobacillus rhamnosus*; LR) on bones in estrogen-deficient rats. The experiments were carried out on mature rats divided into five groups: non-ovariectomized (NOVX) control rats, ovariectomized (OVX) control rats, and OVX rats treated with: LR, AZM, or AZM with LR. The drugs were administered for 4 weeks. Serum biochemical parameters, bone mineralization, histomorphometric parameters, and mechanical properties were examined. Estrogen deficiency increased bone turnover and worsened cancellous bone microarchitecture and mechanical properties. The administration of LR or AZM slightly favorably affected some skeletal parameters of estrogen-deficient rats. The administration of AZM with LR did not lead to the addition of the effects observed for the separate treatments, indicating that the effects could be microbiota-mediated.

## 1. Introduction

Antibiotics may affect the skeletal system directly and/or indirectly by influencing the microbiota. Most of the antibiotics studied so far exert damaging effects on the skeletal system [1]. The exception is tetracyclines; animal studies have shown their beneficial effects on the skeletal system in models of osteoporosis [2,3,4,5,6]. Very little is known about the effects of azithromycin (AZM), a commonly used macrolide antibiotic, on bones.

AZM exerts a broad spectrum of antibacterial activity, mainly against Gram-positive and atypical bacteria. Its bacteriostatic effect results from the inhibition of bacterial protein synthesis by blocking mRNA translation. AZM has a long half-life and large volume of distribution [7,8]. AZM is able to penetrate tissues, including bones [9]. It also accumulates in phagocytes, reaching higher concentrations than in the serum. AZM is mainly used in the treatment of bacterial infections of the respiratory, gastrointestinal, and urogenital systems and the skin [7,8].

AZM also exerts immunomodulatory effects related to the suppression of T lymphocyte activation and the reduction in the release of proinflammatory cytokines and chemokines [7]. The anti-inflammatory effect is mainly due to the inhibition of nuclear factor-кB (NF-кB) activity [7,10]. The immunomodulatory and anti-inflammatory activities of AZM are also used in the long-term treatment of chronic obstructive pulmonary disease, cystic fibrosis, bronchiectasis, bronchiolitis obliterans syndrome, diffuse panbronchiolitis, and asthma [7,11,12,13,14] and in the non-surgical treatment of periodontitis [15,16]. Moreover, some antiviral activity has been attributed to AZM, whose mechanism of action is unclear [17,18]. The antiviral and immunomodulatory activities of AZM were reasons for its use in the treatment of COVID-19 [19,20,21,22,23].

The gut microbiota, containing mostly bacteria belonging to the *Bacteroidetes* and *Firmicutes* phyla, has a large effect on the immune system. The microbiota affects the integrity of the intestinal mucosa, provides essential vitamins and enzymes, protects the body against pathogens, and produces antimicrobial peptides. It also regulates basic human functions, such as digestion, energy metabolism, and inflammation [24,25,26,27,28]. The gut microbiota also affects bone metabolism and some chronic diseases associated with the loss of bone mass and quality, including diabetes, obesity, and inflammatory bowel disease [2,4,28,29,30,31]. Probiotic bacteria have been reported to favorably affect bones in conditions of estrogen deficiency [32,33,34,35,36,37].

Estrogen deficiency associated with menopause is responsible for the disturbance of bone resorption and bone formation. It is the most important risk factor for the development of osteoporosis, characterized by a reduction in bone mass, the deterioration of cancellous and compact bone microstructure, and a decrease in bone strength. Osteoporosis leads to an increase in the bone fracture rate, resulting not only in worsening of life quality but also in increased morbidity and mortality [38,39,40]. The results of the latest studies indicate that, in addition to estrogen deficiency, a key role in the development of osteoporosis is also played by the relationship between the bone and the gut microbiota and the immune system [29,30,41,42,43].

Changes in the gut microbiota are associated with changes in estrogen levels and bone loss. The gut microbiota modulates the inflammation induced by estrogen deficiency and prevents an increased intestinal permeability, and a number of T lymphocytes. Proinflammatory cytokines increase osteoclast formation, activity, and lifespan, which results in increased bone resorption [29,31,32,42,44].

Antimicrobial therapy, especially with broad-spectrum antibiotics, may result in dysbiosis. A typical result of post-antibiotic dysbiosis is the loss of taxonomic and functional variety combined with reduced resistance to invasive pathogens colonization [45,46,47]. AZM, as a broad-spectrum antibiotic, may disturb the gut microbiota and thus indirectly affect the skeletal system. This may be of particular importance in postmenopausal women, whose gut microbiota may be affected by estrogen deficiency. There is also a possibility of a direct effect of AZM on the immune and skeletal systems. Previous studies have indicated that AZM may affect bone directly by inhibiting the formation and resorptive activity of osteoclasts in vitro [48] or indirectly by reducing inflammation associated with pathological bone resorption [49,50,51].

The effects of AZM have never been investigated in conditions of estrogen deficiency. The aim of the present study was to investigate the effects of AZM on the bones of ovariectomized (OVX) rats (a model of postmenopausal osteoporosis). Taking into account its possible effect on the intestinal microflora as a broad-spectrum antibiotic [2,45,46,47,52], we also examined whether the effect of AZM on bone is modified by supplementation with a commonly used probiotic bacteria (*Lactobacillus rhamnosus* (LR)). In addition, the effect of LR alone on the skeletal system of OVX rats was also investigated.

## 2. Results

### 2.1. Effects of AZM and/or LR on Body Mass Gain and Grip Strength

**Figure 1:**
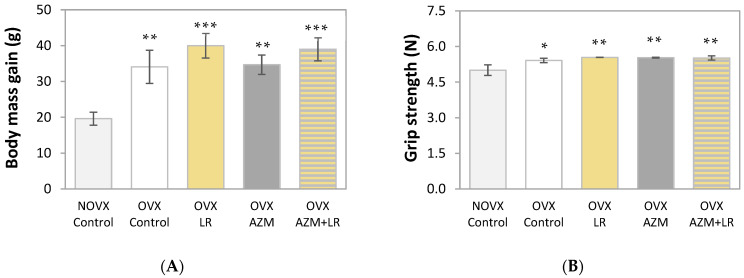
Effect of azithromycin (AZM) and/or *Lactobacillus rhamnosus* (LR) on the body mass gain (**A**) and grip strength (**B**) in ovariectomized rats. The results are presented as means ± SEM. NOVX Control—non-ovariectomized control rats; OVX Control—ovariectomized control rats; OVX LR—OVX rats treated with LR (3 × 10^8^ CFU/kg daily for four weeks); OVX AZM—OVX rats treated with AZM (50 mg/kg daily in the first week and then 3 times a week for three weeks); OVX AZM + LR—OVX rats treated with AZM and LR. The body mass gain occurred during the period of the drug treatment (to the day preceding euthanasia). * *p* < 0.05, ** *p* < 0.01, *** *p* < 0.001—in comparison with the NOVX Control group.

Estrogen deficiency significantly increased body mass gain in relation to non-ovariectomized (NOVX) controls (Figure 1A). The oral administration of AZM alone (50 mg/kg daily in the first week and then three times a week for three weeks), AZM together with LR (3 × 10^8^ colony-forming units (CFU)/kg daily for four weeks), and LR alone did not significantly affect body mass gain in OVX rats.

All estrogen-deficient rats had increased grip strength compared to NOVX controls (Figure 1B), which was independent of the treatment. Neither estrogen deficiency nor the treatments affected the skeletal muscle mass (data not shown).

### 2.2. Effect of AZM and/or LR on Serum Biochemical Parameters

Estrogen deficiency in all groups of OVX rats resulted in increased bone formation (an increased level of a bone formation marker, osteocalcin, and increased activity of alkaline phosphatase (ALP)) and a tendency to increase bone resorption (an insignificant increase in the C-terminal telopeptide fragments of type I collagen (CTX-I) level) in relation to NOVX controls (Table 1). There was no effect of estrogen deficiency on the serum calcium concentration.

The use of AZM alone resulted in a statistically significant increase in the concentration of osteocalcin in relation to OVX control rats; this effect was not demonstrated in rats receiving AZM with LR. There was no effect of AZM on ALP activity (only LR alone significantly decreased ALP activity). The administration of AZM alone and with LR resulted in a statistically significant increase in the concentration of inorganic phosphorus in estrogen-deficient rats. There was no significant effect of AZM administration on bone resorption (the CTX-I concentration did not significantly change). There were no effects of the treatments on hepatic enzyme (alanine aminotransferase (ALT) and aspartate aminotransferase (AST)) activities. Surprisingly, the rats treated with AZM with LR had strongly decreased total protein levels.

### 2.3. Effects of AZM and/or LR on the Mass, Composition, Mineralization, and Density of the Femur

Estrogen deficiency and the treatment of estrogen-deficient rats with AZM and/or LR did not significantly affect bone mass or the mass of bone mineral, organic substances, and water (Table 2; data for the tibia not shown). However, there were significant decreases in the ratios of bone mass and bone mineral mass to body mass in all groups of estrogen-deficient rats compared to NOVX controls.

There were no significant effects of estrogen deficiency or treatments on bone composition (the ratios of the mass of bone mineral, organic substances, and water to bone mass), calcium and phosphorus contents in bone mineral, or the bone density and bone mineral density.

### 2.4. Effect of AZM and/or LR on the Histomorphometric Parameters of The Femur

Estrogen deficiency adversely affected the structure of cancellous bone in the epiphysis and metaphysis of the femur (Table 3). In the OVX control group, there was a statistically significant reduction in the bone volume/tissue volume ratio (BV/TV) and trabecular number (Tb.N) and an increase in trabecular separation (Tb.Sp) in both the epiphysis and metaphysis of the femur in relation to NOVX controls. The value of trabecular thickness (Tb.Th) statistically insignificantly decreased.

The administration of AZM alone exerted a favorable effect on the histomorphometric parameters of cancellous bone in OVX rats. In the epiphysis of the femur, a statistically significant increase in BV/TV and Tb.Th was observed in comparison to OVX control rats. Other parameters also improved; Tb.Sp and Tb.N were no longer significantly different from those of NOVX control rats. Similarly, in the femoral metaphysis, the histomorphometric parameters were slightly improved compared to OVX controls; all of the parameters were no longer significantly different from those of NOVX controls. In contrast, the administration of AZM with LR did not favorably affect the histomorphometric parameters of the femoral epiphysis and metaphysis, similarly to the lack of effect of LR alone.

There were no significant effects of estrogen deficiency or the treatments on the histomorphometric parameters of cortical bone (the femoral diaphysis).

### 2.5. Effects of AZM and/or LR on Bone Mechanical Properties

Estrogen deficiency significantly worsened the strength of the proximal tibial metaphysis (mostly cancellous bone), significantly reducing the values of Young’s modulus, the load, energy, and stress for the yield point, and the load and stress for the maximum load and fracture points in relation to NOVX controls (Table 4). Administration of AZM alone or with LR did not significantly affect the effect of estrogen deficiency on cancellous bone strength. Only for the yield point were favorable effects of LR administration on the mechanical parameters demonstrated (significant increases in the load, displacement, and energy compared to OVX controls). Those effects were completely counteracted by the administration of AZM (the AZM + LR group).

The unfavorable effect of estrogen deficiency on compact bone of the tibial diaphysis was weaker than that on cancellous bone of the tibial metaphysis. There were significant decreases only in the values of stress for the maximum load and fracture points in OVX control rats compared to NOVX control rats (Table 5). All of the treatments significantly improved the mechanical properties of the tibial diaphysis compared to OVX controls; the administration of AZM alone significantly increased the values of the load for the yield point and the maximum load, the administration of AZM with LR significantly increased the values of the maximum load and energy for the fracture point, and the administration of LR alone significantly increased the values of the yield point load and the maximum load.

## 3. Discussion

The results of recent studies indicate a key role of the intestinal microbiota in maintaining the body’s homeostasis, including the control of proper bone remodeling. Disturbance of the composition of the bacterial flora by the use of antibiotics may lead not only to bacterial infections and fungal diseases, or the lack of nutrients produced by bacteria or released by bacterial enzymes, but also to disturbances in the immune response of the body and, consequently, changes in the concentrations of cytokines and growth factors necessary to regulate metabolic processes in bones [2,3,4,31,42,43,53].

The interaction between the gut microbiota and the skeletal system, including the modulation of inflammatory reactions induced by estrogen deficiency, seems well established [31,32,42,43,52,53]. There is an increasing body of data demonstrating that estrogen deficiency leads to changes in the intestinal microbiota, which may contribute to the development of osteoporosis [31,32,34,43,53,54], and that administration of probiotics may exert favorable effects, slowing down the process [32,33,34,35,36,37,54].

Very little is known about the effects of AZM on the skeletal system. According to our knowledge, the effects of long-term AZM treatment on the skeletal system have never been studied. So far, only five reports on the effects of AZM on the skeletal system in humans [51], in rats [49,55], and in vitro [48,56] have been published. There are no experimental or clinical studies on its effects on the development of osteoporosis. However, there is established evidence for an association between exposure to AZM and a reduction in microbiome diversity [47,57,58,59]. The susceptibility to AZM of the LR strains administered in our study was not mentioned in the informatory data from the probiotic producer; however, the LR strains were resistant to erythromycin, another macrolide antibiotic, consistent with the literature data [60,61]. On the other hand, there is a report on the susceptibility of LR strains to both azithromycin and erythromycin [62].

Postmenopausal osteoporosis is the most frequent type of osteoporosis [63]. Estrogen deficiency leads to increases in the bone remodeling rate, with the acceleration of both bone resorption and formation. The prevalence of bone resorption is due to the fact that although estrogen inhibits the formation of both osteoclasts and osteoblasts from their respective progenitors, it concurrently promotes apoptosis in osteoclasts and inhibits apoptosis in osteoblasts and osteocytes [64]. It is thought that the increase in bone resorption correlates not only with the loss of estrogen’s direct effect on osteoclasts but also with the loss of estrogen-dependent immunosuppressive effects resulting in increased production of proinflammatory cytokines that promote osteoclastogenesis, particularly receptor activator of NF-кB ligand (RANKL), tumor necrosis factor α (TNF-α), interleukin-1 (IL-1), and IL-6 [65,66].

In the present study, increases in the biochemical markers of bone turnover were demonstrated in OVX control rats in comparison with NOVX controls, although only the changes in bone formation markers (osteocalcin and ALP) were statistically significant. However, bone loss (decreases in the ratios of bone mass and bone mineral mass to body mass) led to significant decreases in bone strength. The changes in cancellous bone (mechanical properties of the proximal tibial metaphysis and histomorphometric parameters of the femoral epiphysis and metaphysis) were much more significant than those in compact bone (mechanical properties of the tibial diaphysis), consistent with our previous studies [67,68]. Taking into account the abovementioned relations between osteoporosis and changes in the intestinal microbiome, we studied the effects of LR and AZM on the development of skeletal changes in OVX rats.

The results of the present study demonstrated that the administration of LR or AZM improved some investigated parameters, but not necessarily the same ones. The effects of LR and/or AZM on the skeletal system were investigated 5 weeks after the bilateral ovariectomy and after a 4-week treatment period with the drugs. Taking into account the lifespan of rats, a 4-week treatment period in rats corresponds to approximately 2.5 years in humans, and the 5 weeks that elapsed since the ovariectomy correlates with changes that occur in women for about 3 years under estrogen deficiency conditions [69,70]. A period of four weeks was long enough to show the effects induced by treatments with various drugs, as demonstrated in our previous studies [67,71,72,73].

The administration of LR in the present study slightly beneficially affected some parameters concerning bone strength in estrogen-deficient rats. Although there were no effects of LR on bone mass, mineralization, or histomorphometric parameters, an improvement in the mechanical properties of both cancellous bone (increases in the load, displacement, and energy for yield point only in the proximal tibial metaphysis) and compact bone (increases in the yield point load and maximum load in the tibial diaphysis) was noted. These effects may relate to a significant decrease in serum ALP activity. Similar effects on ALP activity were reported for two other *Lactobacillus* species (*Lactobacillus plantarum* and *Lactobacillus fermentum*) administered to OVX rats [74]. However, the lack of an effect on another bone formation marker (osteocalcin) as well as a marker of bone resorption (CTX-I) suggests that mechanisms other than decreasing the accelerated bone turnover may be responsible.

The osteoprotective effect of probiotic administration has been attributed to the inhibition of bone resorption by reducing inflammation, as demonstrated in mouse [75] and rat [76] models of experimental periodontitis, and to the enhancement of bone formation, as shown in mice deficient in sex steroids [32] or in eugonadic mice [77]. The inhibition of bone resorption was also demonstrated in estrogen-deficient mice [32,37]. In OVX mice, LR supplementation decreased intestinal permeability and suppressed inflammation in the intestines and bone marrow, decreased osteoclastogenic cytokine expression, and increased trabecular bone volume [32]. The increase in trabecular bone volume was a result of an increase in bone formation associated with an increase in the osteocalcin level [32] or the induction of T regulatory cells in the intestine and bone marrow, leading to the stimulation of bone formation by activating Wnt signaling in osteoblasts, demonstrated in eugonadic mice [77]. Moreover, in OVX mice, the reduction in bone mass loss induced by probiotic administration correlated with a decrease in the level of osteoclastogenic cytokines (IL-6, IL-17, and TNF-α) with a simultaneous increase in the level of anti-osteoclastogenic cytokines (IL-4, IL-10, and interferon-γ) [37].

The results of the present study indicate that AZM did not unfavorably affect the skeletal system in estrogen-deficient rats. In fact, some results of the study indicate the possibility of its favorable skeletal effects. Although the studies published so far have reported rather a decrease in bone resorption due to AZM [48,49], the results of the present study clearly indicate that AZM may increase bone formation (an increased serum osteocalcin concentration and increases in BV/TV and Tb.Th in the femoral epiphysis). However, the antiresorptive effect of AZM cannot be excluded. Although the mechanical properties of cancellous bone (the proximal tibial metaphysis), which was strongly disordered due to estrogen deficiency, were not improved, significant beneficial effects on the mechanical properties of compact bone (the tibial diaphysis) were demonstrated. On the other hand, after AZM administration, a significant increase in the serum inorganic phosphorus concentration was observed, which may indicate the increased risk of disrupting mineral homeostasis.

As was mentioned before, the results of previous studies indicated the possibility of a decrease in bone resorption due to AZM through the inhibition of inflammation. The ability of AZM to inhibit inflammation, resulting in reduced bone deterioration, has been demonstrated in periodontal inflammation in vitro [48,50,78] and in human studies [51]. AZM also improved trabecular bone microarchitecture in a rat model of experimental periodontitis [49]. The use of AZM resulted in periodontal healing and bone regeneration [51], and its subantibiotic doses reduced alveolar bone destruction and improved trabecular bone microarchitecture [49]. This effect might have been associated with the ability of AZM to inhibit NF-кB [10], which plays an important role in osteoclastogenesis and the development of inflammation [79]. The antiresorptive activity of AZM was also demonstrated in an in vitro study, in which a decrease in osteoclast activity with a decrease in nuclear factor of activated T-cells 1 (NFATc1) expression was noted [48]. The antiresorptive activity of AZM might be connected with a reduction in the expression of proinflammatory cytokines [78]. To our knowledge, the present study is the first report on the influence of AZM on bone formation markers in vivo. So far, it has only been shown that AZM promoted the differentiation of human periodontal ligament stem cells in the inflammatory microenvironment in vitro [50]. However, a recent in vitro study reported that AZM at a high concentration suppressed mineralized nodule formation in osteoblast-like MC3T3-E1 cells [56].

The administration of AZM with LR to OVX rats did not lead to the addition of the effects observed for the separate treatments. Only this group of rats had decreased serum total protein levels. Similarly to the AZM-only-treated rats, favorable effects on the mechanical properties of compact bone and the increased concentration of inorganic phosphorus were observed. However, for the remaining investigated parameters significantly affected by the separate treatments, some antagonistic effects of AZM and LR were observed. In the rats administered AZM and LR, some statistically significant effects demonstrated for LR alone or AZM alone disappeared. In particular, in estrogen-deficient rats, only LR alone decreased the serum ALP activity and favorably affected the mechanical parameters in cancellous bone of the tibial metaphysis, whereas only AZM alone increased the serum concentration of osteocalcin and improved the histomorphometric parameters of the femoral epiphysis (cancellous bone). In addition, increases in the value of the yield point load of the tibial diaphysis (compact bone) were statistically significant in rats receiving LR or AZM but not in rats receiving AZM with LR (on the other hand, favorable effects of all treatments were observed for the maximum load).

The reason for the reciprocal attenuation of the effects of both AZM and LR after their combined use in OVX rats may be only speculated. The decrease in the LR effect caused by AZM may have resulted from its susceptibility to AZM [62], but the interpretation of the decrease in the AZM effect caused by LR is more difficult. The differential effects might have resulted from the influence on the gut microbiota. It is possible that changes in the gut microbiota leading to changes in cytokine expression may have exerted differential effects on the activity of osteoblasts. Interestingly, two serum markers of bone formation, ALP and osteocalcin, were differentially affected by the separate treatments, and there was no effect on these parameters in the combined treatment (AZM alone increased the level of osteocalcin, LR alone decreased the ALP activity, and AZM with LR had no effect).

## 4. Materials and Methods

### 4.1. Animals and Drugs Used

Three-month-old female Wistar rats obtained from the Center of Experimental Medicine, Medical University of Silesia, Katowice, Poland, were used in the study. The animals were fed a standard laboratory diet (Labofeed B, Wytwórnia Pasz “Morawski”, Kcynia, Poland) ad libitum and had unrestricted access to water during the 7-day acclimatization period and throughout the experiment. The rats were maintained under monitored standard laboratory conditions, complying with the European Union guidelines (Directive 2010/63/EU). All procedures of the experiments on animals were approved by the Local Ethics Committee, Katowice, Poland (approval nos.: 76/2018 and 43/2019).

Drugs used in the study: azithromycin tablets 500 mg (Sumamed, Teva Pharmaceuticals Polska Sp. z o.o., Warszawa, Poland), probiotic *Lactobacillus rhamnosus* powder for oral suspension minimum 10^10^ colony forming units (CFU) (Lakcid forte, Biomed-Lublin Wytwórnia Surowic i Szczepionek S.A., Lublin, Poland; a mixture of 3 LR strains: Pen, E/N, and Oxy), and drugs used for general anesthesia during ovariectomy and euthanasia: ketamine (Ketamina 100 mg/mL, Biowet Puławy Sp. z o.o., Puławy, Poland) and xylazine (Sedazin, Biowet Puławy Sp. z o.o., Puławy, Poland).

After 7 days of acclimatization, the rats were divided into five groups (NOVX Control group n = 11, OVX Control group n = 12, and the remaining groups n = 10):

1. NOVX control rats;
2. OVX control rats;
3. OVX rats treated with LR;
4. OVX rats treated with AZM;
5. OVX rats treated with AZM and LR.

The bilateral ovariectomy was performed in rats from groups 2–5 under general anesthesia induced by intraperitoneal injections of a mixture of ketamine and xylazine. The administration of AZM and the probiotic started 7 days after the surgery. The average body mass in the investigated groups of rats at the start of the drug administration, 7 days after the ovariectomy, ranged from 184.2 g to 199.3 g. There were no statistically significant differences in the initial body mass between the groups. The drugs were administered orally (by oral gavage) for 4 weeks: AZM (at a dose of 50 mg/kg) was administered once daily in the first week of the experiment and then 3 times a week (every second or third day), and LR (at a dose of 3 × 10^8^ CFU/kg) was administered once daily throughout the experiment. The AZM dose was calculated [80] based on its antimicrobial dose used in patients and the antibiotic AZM dose, reported to affect the rat skeletal system [49], taking into consideration its bioavailability in rats [81]. The dose of the probiotic was chosen based on previous studies [82,83].

To adjust the dose to increasing body mass, the rats were weighed at the start of the experiment and then once a week. Moreover, taking into account bone–muscle interactions [84], the grip strength of the forelimbs (peak force) was determined on the last day of drug administration using a grip strength meter apparatus for rats and mice (model 47200; Ugo Basile, Gemonio Italy), as previously described [72]. Data were monitored, transferred, and analyzed on a computer with the use of data collection application (DCA) software version 2.2 (Ugo Basile, Gemonio, Italy).

After the 4-week treatment, the rats were fasted overnight with ad libitum access to water and then were euthanized under general anesthesia (induced by a mixture of ketamine and xylazine injected intraperitoneally) by cardiac exsanguination. After the vital functions had ceased, bones (left and right femurs and tibias) and muscles (musculus gastrocnemius, musculus soleus, and musculus tibialis anterior) were isolated, and blood was taken for further biochemical tests. The bones were cleaned of soft tissues. The left femurs and tibias were wrapped in gauze, soaked in 0.9% sodium chloride solution, and placed at −18 °C until used for further studies. The blood was centrifuged, and the serum was frozen at −80 °C until used for biochemical studies.

### 4.2. Biochemical Studies

The levels of bone markers: the bone resorption marker, C-terminal telopeptide fragments of type I collagen (RatLaps CTX-I EIA), and the bone formation marker, osteocalcin (Rat-MID Osteocalcin EIA), were determined with the use of ELISA kits (Immunodiagnostic Systems Ltd., Boldon, UK). Spectrophotometric measurements were performed with the use of a Tecan Infinite M200 Pro microplate reader with attached Magellan 7.2 software (Tecan Austria, Grödig, Austria).

In addition, the concentration of calcium and inorganic phosphorus and the activity of ALP in the serum were measured using an automatic biochemical analyzer (Mindray BS-240, Shenzhen, China) with commercially available kits (Pointe Scientific, Canton, MI, USA).

To determine the possible hepatotoxic effects of AZM, liver enzyme (AST and ALT) activities and serum total protein levels were determined using an automatic biochemical analyzer (Mindray BS-240, Shenzhen, China) with commercially available kits (Pointe Scientific, Canton, MI, USA).

### 4.3. Bone Macrometric Parameters, Composition, and Mineralization Studies

After cleaning the left bones of soft tissues, the diameters (determined in the middle of the bone) and bone lengths were measured with the use of a digital caliper (Topex, Warszawa, Poland). The bones were weighed on an Adventurer Pro type AV264CM analytical balance (Ohaus Europe GmbH, Greifensee, Switzerland).

In order to remove water from the left femurs and tibias, bones were lyophilized for 11 days at a temperature of −51 °C and pressure of 0.03 mBa in the lyophilizer FreeZone 6 (Labconco, Kansas City, MO, USA). The bone mass after lyophilization was determined. The bones were then ashed (mineralization process to remove organic components from bones) at a temperature of 640 °C for 48 h in a muffle furnace L9/11/C6 (Nabertherm, Lilienthal, Germany). Ashed bones were weighed to determine the bone mineral mass. The bone water mass was calculated by subtracting the bone mass after lyophilization from the bone mass. The bone organic substance mass was determined by subtracting the mass of bone mineral from the bone mass after lyophilization. The bone content of mineral was calculated as the ratio of the mass of bone mineral to the bone mass. The bone content of organic substances was calculated as the ratio of the mass of bone organic substances to the bone mass. The bone content of water was calculated as the ratio of the mass of bone water to the bone mass. Moreover, the ratio of bone mass to body mass before overnight fasting and the ratio of bone mineral mass to body mass before overnight fasting were calculated.

The ashed bones were dissolved in 6 M hydrochloric acid and then diluted in deionized water to prepare a solution for measuring calcium and phosphorus concentrations using an automatic biochemical analyzer (Mindray BS-240, Shenzhen, China) with commercially available kits (Pointe Scientific, Canton, MI, USA). The calcium and phosphorus contents in bone mineral were calculated based on the concentrations measured.

### 4.4. Bone Histomorphometric Studies

The histomorphometric measurements of the longitudinal cross-sections of the distal femoral metaphysis and epiphysis were performed on decalcified preparations that were stained with hematoxylin and eosin, prepared as previously described [68,71,72], and the measurements of the transverse cross-sections of the femoral diaphysis were made on undecalcified, unstained slides of the femoral diaphysis, prepared as previously described [5,68,71,72].

The measurements of histomorphometric parameters were performed with the use of the OsteoMeasure system consisting of an Axio Imager.A1 microscope (Carl Zeiss, Göttingen, Germany) connected to an Olympus DP71 camera (Olympus, Tokyo, Japan), Cintiq 22HD graphic tablet (Wacom, Kazo, Japan) and computer with OsteoMeasure XP v1.3.0.1 software (OsteoMetrics, Decatur, GA, USA). The description of the studied parameters was made using the standardized nomenclature for bone histomorphometry developed by the American Society for Bone and Mineral Research (ASBMR) [85].

In cancellous bone (distal femoral metaphysis and epiphysis), the following histomorphometric parameters were measured: BV/TV, Tb.Th, Tb.Sp, and Tb.N.

The following histomorphometric parameters were determined in cortical bone (femoral diaphysis): transverse cross-sectional area of the cortical area (Ct.Ar), transverse cross-sectional area of the marrow cavity (Ma.Ar), transverse cross-sectional area of the total diaphysis (Tt.Ar), and transverse cross-sectional area of the marrow cavity/total diaphysis area ratio (Ma.Ar/Tt.Ar).

### 4.5. Bone Mechanical Property Studies

The mechanical properties of the proximal tibial metaphysis and the tibial diaphysis were performed using an Instron 3342 500N apparatus (Instron, Norwood, MA, USA) and Bluehill 2.14 software for data analysis. In the three-point bending tests (displacement rate of 0.01 mm/s; sampling frequency of 100 Hz) conducted in the left tibias, extrinsic parameters (load, displacement, and energy for the yield point (0.05% offset), maximum load point, and fracture point) and intrinsic parameters (stress for the yield point (0.05% offset), maximum load point, and fracture point and Young’s modulus) were determined. The yield point is the force limit above which the bone structure becomes permanently damaged [86]. The maximum load is the ultimate load the bone can sustain, and the fracture load is the load registered at the bone fracture.

To determine the mechanical properties of the proximal tibial metaphysis, the epiphysis was removed before the test, and the test was performed as previously described [67,68,87]. The support points were the edge of the proximal tibial metaphysis and the location of the fibula attachment to the tibial bone. The distance between these points was measured with a digital caliper (Topex, Warszawa, Poland) each time. The load was applied perpendicularly to the long axis of the tibia, 3 mm from the edge of the proximal tibial metaphysis. The test was preceded by applying a preload of 1 N to stabilize the bone. In order to estimate the moment of inertia, which is required for the calculations of stress and Young’s modulus values, it was assumed that the cross-section of the proximal tibial metaphysis at the fracture site has the shape of a circular beam. The cross-sectional diameter was measured using a digital caliper (Topex, Warszawa, Poland).

After performing the test on the proximal metaphysis, the strength parameters of the tibial diaphysis were measured [86]. The tibia was placed on two supports, 16 mm apart. The load was placed in the middle of the tibial bone, perpendicularly to the longitudinal axis of the bone. A preload of 1 N was applied to stabilize the bone before the proper test. To determine the moment of inertia in the fracture area, which is necessary for the calculations of values of stress and Young’s modulus, it was assumed that the tibial diaphysis at the fracture site has the shape of a circular beam (the diameter in the middle of the tibia was measured with a digital caliper).

### 4.6. Statistical Analysis

The results of the present study are presented as the mean ± standard error of the mean (SEM). Statistical significance of the results was assessed using one-way analysis of variance (ANOVA), followed by Fisher’s Least Significant Difference (LSD) post hoc test (Statistica 13.3; Tibco Software Inc., Palo Alto, CA, USA). The results obtained in OVX rats treated with LR (OVX LR group), AZM (OVX AZM group), and AZM with LR (OVX AZM + LR group) were compared with those of OVX control rats (OVX Control group). Moreover, results obtained in rats receiving AZM with LR (OVX AZM + LR group) were compared with those of rats administered AZM or LR alone. The results from all OVX groups were compared with those from NOVX control rats (NOVX Control group). The results were assumed to be statistically significant if *p* < 0.05.

## 5. Conclusions

In conclusion, long-term use of AZM had a beneficial effect on bones in estrogen-deficient rats, which was decreased by concomitant *Lactobacillus rhamnosus* administration, indicating that the effect of AZM was dependent on the composition of the intestinal microflora.

## Figures and Tables

**Table 1:** Effect of azithromycin (AZM) and/or *Lactobacillus rhamnosus* (LR) on serum biochemical parameters in ovariectomized rats.

Parameter	NOVX Rats	OVX Rats			
	Control	Control	LR	AZM	AZM + LR
CTX-I (ng/mL)	21.73 ± 1.86	26.34 ± 2.03	27.42 ± 2.04	30.52 ± 2.47	26.83 ± 2.91
Osteocalcin (ng/mL)	339.46 ± 15.97	458.87 ± 21.82 **	491.08 ± 35.93 ***	553.56 ± 31.28 *** ^●^	494.98 ± 27.13 ***
ALP (U/L)	71.35 ± 5.59	112.24 ± 8.80 ***	91.38 ± 4.58 * ^●^	108.84 ± 7.07 ***	101.04 ± 5.03 **
Calcium (mg/dL)	11.05 ± 0.13	11.13 ± 0.14	11.19 ± 0.09	11.10 ± 0.24	10.90 ± 0.16
Inorganic phosphorus (mg/dL)	6.45 ± 0.26	6.23 ± 0.28	6.79 ± 0.26	7.17 ± 0.29 ^●^	7.49 ± 0.26 ** ^●●^
Total protein (g/dL)	5.20 ± 0.11	5.14 ± 0.09	5.07 ± 0.11	4.93 ± 0.08	4.65 ± 0.10 *** ^●●● ##^
ALT (U/L)	50.03 ± 7.66	53.12 ± 5.22	49.69 ± 4.05	48.73 ± 3.56	56.87 ± 5.99
AST (U/L)	168.15 ± 18.84	163.11 ± 17.58	198.77 ± 15.20	191.77 ± 14.88	206.33 ± 19.50

The results are presented as means ± SEM. NOVX Control—non-ovariectomized control rats; OVX Control—ovariectomized control rats; OVX LR—OVX rats treated with LR (3 × 10^8^ CFU/kg daily for four weeks); OVX AZM—OVX rats treated with AZM (50 mg/kg daily in the first week and then 3 times a week for three weeks); OVX AZM + LR—OVX rats treated with AZM and LR. CTX-I—C-terminal telopeptide fragments of type I collagen; ALP—alkaline phosphatase; ALT—alanine aminotransferase; AST—aspartate aminotransferase. * *p* < 0.05, ** *p* < 0.01, *** *p* < 0.001—in comparison with the NOVX Control group. ^●^ *p* < 0.05, ^●●^ *p* < 0.01, ^●●●^ *p* < 0.001—in comparison with the OVX Control group. ^##^ *p* < 0.01—in comparison with the OVX LR group.

**Table 2:** Effect of azithromycin (AZM) and/or *Lactobacillus rhamnosus* (LR) on the mass, composition, mineralization, and density of the femur in ovariectomized rats.

Parameter	NOVX Rats	OVX Rats			
	Control	Control	LR	AZM	AZM + LR
Bone mass (g)	0.616 ± 0.016	0.622 ± 0.012	0.650 ± 0.013	0.626 ± 0.017	0.623 ± 0.011
Bone mass/body mass ratio (g/100 g) ^&^	0.302 ± 0.004	0.274 ± 0.012 **	0.274 ± 0.007 *	0.268 ± 0.006 **	0.268 ± 0.006 **
Bone mineral mass (g)	0.284 ± 0.006	0.276 ± 0.004	0.289 ± 0.007	0.285 ± 0.007	0.282 ± 0.007
Bone organic substance mass (g)	0.147 ± 0.004	0.147 ± 0.002	0.155 ± 0.003	0.151 ± 0.004	0.149 ± 0.002
Bone water mass (g)	0.185 ± 0.007	0.199 ± 0.013	0.205 ± 0.006	0.191 ± 0.006	0.192 ± 0.003
Bone mineral mass/ body mass ratio (g/100 g) ^&^	0.139 ± 0.001	0.121 ± 0.003 ***	0.122 ± 0.002 ***	0.122 ± 0.003 ***	0.121 ± 0.003 ***
Mineral mass/bone mass ratio (g/g)	0.461 ± 0.004	0.445 ± 0.009	0.445 ± 0.006	0.455 ± 0.004	0.452 ± 0.004
Organic substance mass/ bone mass ratio (g/g)	0.239 ± 0.001	0.238 ± 0.005	0.239 ± 0.003	0.241 ± 0.002	0.239 ± 0.002
Water mass/bone mass ratio (g/g)	0.300 ± 0.005	0.318 ± 0.013	0.316 ± 0.005	0.304 ± 0.004	0.308 ± 0.004
Calcium content (g/g of bone mineral)	0.371 ± 0.006	0.368 ± 0.006	0.364 ± 0.003	0.363 ± 0.005	0.367 ± 0.005
Phosphorus content (g/g of bone mineral)	0.143 ± 0.003	0.143 ± 0.002	0.143 ± 0.002	0.142 ± 0.003	0.142 ± 0.001
Bone density (g/cm^3^)	1.606 ± 0.007	1.586 ± 0.007	1.573 ± 0.012	1.585 ± 0.007	1.584 ± 0.009
Bone mineral density (g/cm^3^)	0.716 ± 0.009	0.690 ± 0.009	0.675 ± 0.015	0.690 ± 0.008	0.688 ± 0.013

The results are presented as means ± SEM. NOVX Control—non-ovariectomized control rats; OVX Control—ovariectomized control rats; OVX LR—OVX rats treated with LR (3 × 10^8^ CFU/kg daily for four weeks); OVX AZM—OVX rats treated with AZM (50 mg/kg daily in the first week and then 3 times a week for three weeks); OVX AZM + LR—OVX rats treated with AZM and LR. ^&^—Body mass on the day preceding euthanasia. * *p* < 0.05, ** *p* < 0.01, *** *p* < 0.001—in comparison with the NOVX Control group.

**Table 3:** Effect of azithromycin (AZM) and/or *Lactobacillus rhamnosus* (LR) on the histomorphometric parameters of the femur in ovariectomized rats.

Bone	Parameter	NOVX Rats	OVX Rats			
		Control	Control	LR	AZM	AZM + LR
Femoral diaphysis	Ct.Ar (mm^2^)	4.77 ± 0.05	4.94 ± 0.10	5.08 ± 0.16	5.05 ± 0.09	4.89 ± 0.08
	Ma.Ar (mm^2^)	2.78 ± 0.12	2.61 ± 0.08	2.58 ± 0.07	2.70 ± 0.11	2.64 ± 0.08
	Tt.Ar (mm^2^)	7.55 ± 0.13	7.54 ± 0.13	7.65 ± 0.19	7.75 ± 0.17	7.53 ± 0.11
	Ma.Ar/Tt.Ar	0.367 ± 0.010	0.346 ± 0.008	0.337 ± 0.009	0.347 ± 0.009	0.351 ± 0.008
Femoral epiphysis	BV/TV (%)	30.04 ± 1.50	25.18 ± 1.63 *	23.77 ± 1.22 **	31.30 ± 2.15 ^●●^	24.93 ± 1.30 * ^^
	Tb.Th (μm)	63.44 ± 3.66	58.61 ± 3.21	55.31 ± 2.24	70.75 ± 3.69 ^●●^	61.66 ± 2.51
	Tb.Sp (μm)	148.88 ± 7.28	176.40 ± 7.68 *	179.65 ± 8.08 *	159.22 ± 11.02	189.61 ± 11.41 ** ^
	Tb.N (1/mm)	4.79 ± 0.19	4.29 ± 0.12 *	4.30 ± 0.16 *	4.41 ± 0.17	4.04 ± 0.15 **
Femoral metaphysis	BV/TV (%)	32.09 ± 1.52	25.65 ± 1.34 **	26.02 ± 1.83 *	28.27 ± 1.35	25.01 ± 2.02 **
	Tb.Th (μm)	55.42 ± 2.11	53.71 ± 2.84	51.46 ± 2.33	54.84 ± 2.59	53.45 ± 2.76
	Tb.Sp (μm)	119.75 ± 7.26	158.71 ± 10.34 **	152.83 ± 14.67 *	141.11 ± 7.66	167.49 ± 13.04 **
	Tb.N (1/mm)	5.83 ± 0.28	4.84 ± 0.24 **	5.05 ± 0.28	5.22 ± 0.28	4.67 ± 0.29 **

The results are presented as means ± SEM. NOVX Control—non-ovariectomized control rats; OVX Control—ovariectomized control rats; OVX LR—OVX rats treated with LR (3 × 10^8^ CFU/kg daily for four weeks); OVX AZM—OVX rats treated with AZM (50 mg/kg daily in the first week and then 3 times a week for three weeks); OVX AZM + LR—OVX rats treated with AZM and LR. Ct.Ar—transverse cross-sectional area of the cortical area; Ma.Ar—transverse cross-sectional area of the marrow cavity; Tt.Ar—transverse cross-sectional area of the total diaphysis; Ma.Ar/Tt.Ar—transverse cross-sectional area of the marrow cavity/total diaphysis area ratio; BV/TV—bone volume/tissue volume ratio; Tb.Th—trabecular thickness; Tb.Sp—trabecular separation; Tb.N—trabecular number. * *p* < 0.05, ** *p* < 0.01—in comparison with the NOVX Control group. ^●●^ *p* < 0.01—in comparison with the OVX Control group. ^ *p* < 0.05, ^^ *p* < 0.01—in comparison with the OVX AZM group.

**Table 4:** Effect of azithromycin (AZM) and/or *Lactobacillus rhamnosus* (LR) on mechanical properties of the proximal tibial metaphysis in ovariectomized rats.

Parameter	NOVX Rats	OVX Rats			
	Control	Control	LR	AZM	AZM + LR
Young’s modulus (MPa)	3284 ± 336	2354 ± 149 *	1974 ± 216 **	2561 ± 374	2447 ± 378
Yield point load (N)	57.8 ± 9.3	33.3 ± 2.5 ***	48.2 ± 3.5 ^●^	41.6 ± 3.1 *	30.7 ± 3.4 *** ^#^
Displacement for yield point load (mm)	0.39 ± 0.07	0.25 ± 0.02	0.51 ± 0.08 ^●●●^	0.31 ± 0.02	0.26 ± 0.03 ^##^
Energy for yield point load (mJ)	12.8 ± 4.5	4.4 ± 0.5 **	11.8 ± 2.3 ^●^	6.0 ± 0.7 *	4.2 ± 0.8 * ^#^
Stress for yield point load (MPa)	47.5 ± 8.0	26.4 ± 2.1 ***	33.4 ± 8.4 *	30.1 ± 2.9 **	23.4 ± 3.3 ***
Maximum load (N)	101.6 ± 6.6	61.3 ± 2.7 ***	65.6 ± 3.0 ***	65.9 ± 2.5 ***	63.5 ± 5.1 ***
Displacement for maximum load (mm)	0.78 ± 0.07	0.83 ± 0.04	0.94 ± 0.06	0.79 ± 0.05	0.87 ± 0.05
Energy for maximum load (mJ)	42.6 ± 4.8	33.8 ± 2.5	36.9 ± 3.8	32.1 ± 1.6	35.1 ± 3.1
Stress for maximum load (MPa)	83.8 ± 7.5	48.8 ± 2.7 ***	45.1 ± 1.6 ***	48.2 ± 2.8 ***	47.6 ± 4.6 ***
Fracture load (N)	70.9 ± 5.6	47.2 ± 2.1 ***	52.8 ± 3.0 **	48.0 ± 2.7 ***	54.1 ± 4.6 **
Displacement for fracture load (mm)	1.09 ± 0.09	1.25 ± 0.07	1.34 ± 0.08	1.19 ± 0.05	1.19 ± 0.06
Energy for fracture load (mJ)	68.8 ± 6.8	56.7 ± 3.7	59.2 ± 3.9	55.6 ± 2.9	52.7 ± 3.3
Stress for fracture load (MPa)	58.8 ± 6.2	37.9 ± 2.7 ***	36.5 ± 2.2 ***	35.4 ± 2.8 ***	41.1 ± 4.6 **

The results are presented as means ± SEM. NOVX Control—non-ovariectomized control rats; OVX Control—ovariectomized control rats; OVX LR—OVX rats treated with LR (3 × 10^8^ CFU/kg daily for four weeks); OVX AZM—OVX rats treated with AZM (50 mg/kg daily in the first week and then 3 times a week for three weeks); OVX AZM + LR—OVX rats treated with AZM and LR. * *p* < 0.05, ** *p* < 0.01, *** *p* < 0.001—in comparison with the NOVX Control group. ^●^ *p* < 0.05, ^●●●^ *p* < 0.001—in comparison with the OVX Control group. ^#^ *p* < 0.05, ^##^ *p* < 0.01—in comparison with the OVX LR group.

**Table 5:** Effect of azithromycin (AZM) and/or *Lactobacillus rhamnosus* (LR) on mechanical properties of the tibial diaphysis in ovariectomized rats.

Parameter	NOVX Rats	OVX Rats			
	Control	Control	LR	AZM	AZM + LR
Young’s modulus (MPa)	12,587 ± 953	11,288 ± 685	11,039 ± 707	10,396 ± 502	11,328 ± 396
Yield point load (N)	54.7 ± 5.2	54.1 ± 3.8	63.5 ± 1.0 ^●^	65.8 ± 2.0 * ^●^	60.6 ± 1.6
Displacement for yield point load (mm)	0.26 ± 0.02	0.25 ± 0.02	0.29 ± 0.01	0.30 ± 0.01	0.27 ± 0.00
Energy for yield point load (mJ)	7.7 ± 0.9	7.2 ± 0.8	9.2 ± 0.3	9.3 ± 0.4	8.2 ± 0.3
Stress for yield point load (MPa)	175.1 ± 19.0	151.4 ± 11.1	172.4 ± 8.6	163.4 ± 5.1	166.2 ± 5.3
Maximum load (N)	70.5 ± 3.0	64.3 ± 3.7	73.7 ± 2.3 ^●^	76.1 ± 2.7 ^●●^	73.4 ± 1.6 ^●^
Displacement for maximum load (mm)	0.41 ± 0.01	0.40 ± 0.03	0.42 ± 0.03	0.41 ± 0.02	0.44 ± 0.02
Energy for maximum load (mJ)	16.6 ± 3.8	16.8 ± 2.0	18.7 ± 2.2	17.3 ± 1.0	20.0 ± 1.2
Stress for maximum load (MPa)	217.8 ± 9.0	179.7 ± 11.2 **	198.5 ± 7.7	188.4 ± 4.6 *	201.3 ± 6.2
Fracture load (N)	57.0 ± 5.3	48.9 ± 3.6	56.3 ± 4.2	55.5 ± 3.9	52.4 ± 2.8
Displacement for fracture load (mm)	0.74 ± 0.07	0.72 ± 0.05	0.82 ± 0.05	0.78 ± 0.06	0.92 ± 0.08
Energy for fracture load (mJ)	35.7 ± 3.6	34.1 ± 3.3	42.1 ± 2.1	39.9 ± 3.3	49.2 ± 5.2 * ^●●^
Stress for fracture load (MPa)	171.7 ± 8.9	136.4 ± 10.7 **	149.3 ± 7.5	135.8 ± 5.2 **	143.1 ± 6.6 *

The results are presented as means ± SEM. NOVX Control—non-ovariectomized control rats; OVX Control—ovariectomized control rats; OVX LR—OVX rats treated with LR (3 × 10^8^ CFU/kg daily for four weeks); OVX AZM—OVX rats treated with AZM (50 mg/kg daily in the first week and then 3 times a week for three weeks); OVX AZM + LR—OVX rats treated with AZM and LR. * *p* < 0.05, ** *p* < 0.01—in comparison with the NOVX Control group. ^●^ *p* < 0.05, ^●●^ *p* < 0.01—in comparison with the OVX Control group.

## Data Availability

Data is contained within the article.
